# DDX3, a potential target for cancer treatment

**DOI:** 10.1186/s12943-015-0461-7

**Published:** 2015-11-05

**Authors:** Guus Martinus Bol, Min Xie, Venu Raman

**Affiliations:** Department of Pathology, University Medical Center Utrecht Cancer Center, 3508 GA Utrecht, The Netherlands; Department of Radiology and Radiological Science, Johns Hopkins University School of Medicine, 720 Rutland Ave, Traylor 340, Baltimore, MD 21205 USA; Department of Oncology, Johns Hopkins University School of Medicine, Baltimore, MD USA

**Keywords:** DDX3, RNA helicase, Cancer, Small molecule inhibitor, Radiation sensitizing agents

## Abstract

RNA helicases are a large family of proteins with a distinct motif, referred to as the DEAD/H (Asp-Glu-Ala-Asp/His). The exact functions of all the human DEAD/H box proteins are unknown. However, it has been consistently demonstrated that these proteins are associated with several aspects of energy-dependent RNA metabolism, including translation, ribosome biogenesis, and pre-mRNA splicing. In addition, DEAD/H box proteins participate in nuclear-cytoplasmic transport and organellar gene expression.

A member of this RNA helicase family, DDX3, has been identified in a variety of cellular biogenesis processes, including cell-cycle regulation, cellular differentiation, cell survival, and apoptosis. In cancer, DDX3 expression has been evaluated in patient samples of breast, lung, colon, oral, and liver cancer. Both tumor suppressor and oncogenic functions have been attributed to DDX3 and are discussed in this review. In general, there is concordance with *in vitro* evidence to support the hypothesis that DDX3 is associated with an aggressive phenotype in human malignancies. Interestingly, very few cancer types harbor mutations in DDX3, which result in altered protein function rather than a loss of function.

Efficacy of drugs to curtail cancer growth is hindered by adaptive responses that promote drug resistance, eventually leading to treatment failure. One way to circumvent development of resistant disease is to develop novel drugs that target over-expressed proteins involved in this adaptive response. Moreover, if the target gene is developmentally regulated, there is less of a possibility to abruptly accumulate mutations leading to drug resistance. In this regard, DDX3 could be a druggable target for cancer treatment. We present an overview of DDX3 biology and the currently available DDX3 inhibitors for cancer treatment.

## Background

The secondary and tertiary structure of RNA and its interaction with other proteins are important for the function of RNA and the cell as a whole. This process is heavily regulated by chaperones like RNA helicases, which are able to unwind RNA duplexes or displace bound proteins in an energy-dependent fashion. RNA metabolism by helicases is essential in processes such as transcription, ribosome biogenesis, splicing, RNA editing, RNA export from the nucleus, translation initiation, and RNA turnover [1]. The largest group of RNA helicases is the DEAD-box proteins, which belong to the helicase superfamily 2. These DEAD-box helicases are named after the conserved amino acid sequence DEAD (Asp-Glu-Ala-Asp) and are characterized by 12 conserved motifs [2]. The non-conserved domains at the N- and C-terminus largely determine the specific interactions, sub-cellular localization, and expression patterns of each DEAD-box helicase [3]. DEAD-box helicases are present in almost all organisms, conserved from human to yeast, and play a crucial role, as knockdown of these helicases are embryonically lethal [4].

**Fig. 1:**
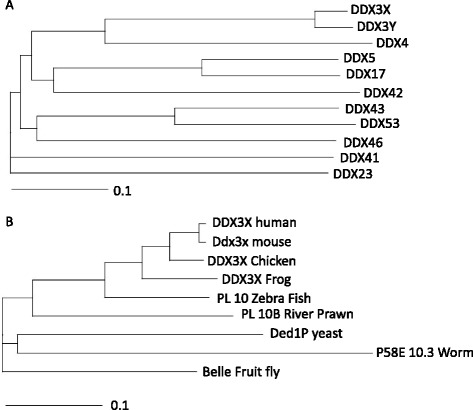
Phylogenetic tree depicting homologous of DDX3. **a**. Phylogram of human DDX3 homologous (Ded1/P68 cluster) made in clustalX (guide tree). **b.** Phylogram of DDX3 orthologs in commonly used model organisms made in clustalX (guide tree)

DDX3 is a highly conserved subfamily of the DEAD-box proteins. There are 37 different DEAD-box proteins in humans, of which the most similar to DDX3 (DED1) are shown in Fig. 1a [5]. In humans, there are at least two pseudo genes and two DDX3 homologs, DDX3X and DDX3Y [6]. Although DDX3X and DDX3Y share 92 % protein sequence identity, they have very different functions and expression patterns in various organs. DDX3Y is located in the azoospermia factor a (AZFa) region of the Y-chromosome, is only expressed in the testes, and plays an important role in male fertility. Deletion of DDX3Y causes azoospermia and cannot be rescued by the DDX3X homologue in humans [7, 8].

DDX3X on the other hand is located on the X-chromosome bands p11.3-- > p11.23 [9] and is ubiquitously transcribed in all human tissues. Deletion of DDX3 is embryonically lethal; however, Ded1P (Yeast orthologue) deletion can be rescued by human DDX3 [10] or Belle (Drosophila orthologue) [11], which underscores the conserved functionality across different species (Fig. 1b). Mice, on the other hand, have three DDX3 homologues - Ddx3x, Dby, and PL10 - which together have functions similar to those of the two human homologues [12–14].

Functionally, DDX3 appears to be one of the most multifaceted helicases with various roles in immunology and cancer. The function of DDX3 in viral manipulation has been extensively reviewed [15], but its importance in cancer is a more recent advancement in science and will be the focus of this comprehensive review.

### DDX3X structure

**Fig. 2:**
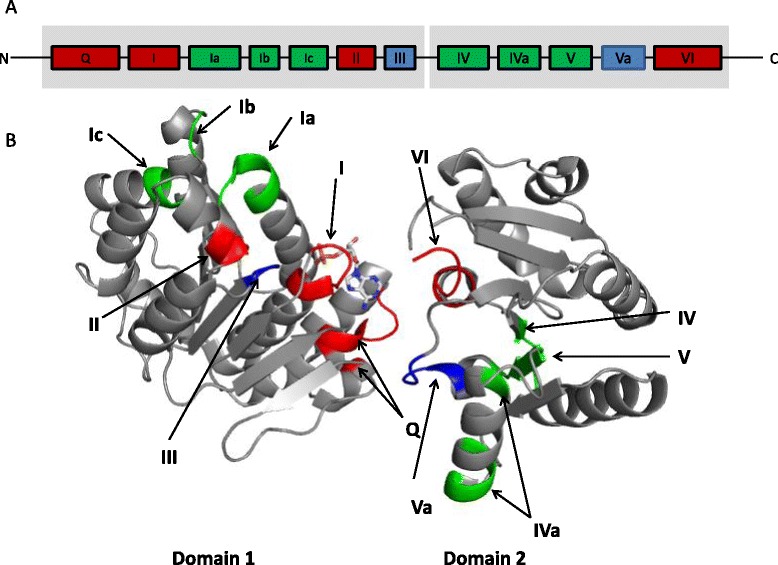
Structure of RNA helicase DDX3. **a.** Schematic representation of DDX3 (human) and conserved motifs. In grey the two RecA-like domains. The motifs include Q (^182^F--^200^YTRPTPVQ), I (^226^TGSGKT), Ia (^274^PTRELA), Ib (^302^GG), Ic (^323^TPGR), II (^347^DEAD), III (^382^SAT), IV (^445^LVFVET), Iva (^477^QRDR--^487^F), V (^494^ILVAT), Va (^502^ARGLD), VI (^527^HRIGRTGR). Conserved amino acid sequences are indicated in parenthesis. Boxes represent the conserved motifs involved in ATP binding (red), RNA binding (green) and linking (blue). **b.** Crystallography structure of DDX3 (V168-G582) (PDB: 2I4I) with AMP as the substrate (12 conserved motifs are indicated with colors)

**Fig. 3:**
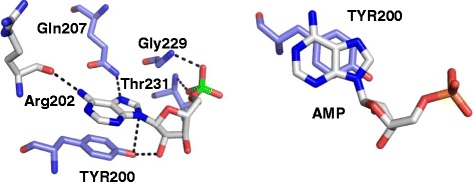
DDX3 interactions with AMP. **a.** Hydrogen interactions between AMP and amino acid residues of DDX3 ATP binding pocket: the C6 amino group of AMP as a hydrogen donor (HD) and the backbone carbonyl oxygen of Arg202 as a hydrogen acceptor (HA); the 2’-OH group as well as N9 of AMP (both act as HA) and the phenolic oxygen of Tyr200 (HD); N7 of AMP (HA) and the side chain NH2 group of Gln207 (HD); two phosphate oxygens of AMP (HA) and the backbone NH groups of Gly229 and Thr 231 (HD). **b.** π-π interaction between the aromatic ring of AMP and the phenol side chain of Tyr200

Like other members of the DEAD-box helicase family, DDX3 consists of two recA-like domains and 12 conserved motifs (Fig. 2a). DDX3X structure has been studied by protein crystallography and X-ray diffraction [16, 17]. A smaller fraction (V168-G582) of the 662 amino acid DDX3X protein was co-crystallized with ATPγS and ADP by Högbom et.al., which resulted in a crystal structure of DDX3X with AMP (Fig. 2b) [17]. DDX3X with 12 conserved motifs (shown in Fig. 2b) has highly similar interaction with AMP to the *thermophilus* DEAD-box helicase Hera. The interaction of AMP with amino acid residues in the nucleotide-binding pocket of DDX3X (V168-G582) is shown in Fig. 3. Purine nucleobase stacks over phenyl group of Tyr 200. The adenine moiety of AMP interacts with amino acids in the Q motif (Arg 202 and Gln 207), whereas residues in the P-loop in motif I interact with the phosphate group (Gly 227, Ser228, Gly 229, Lys 230 and Thr 231).

The lack of ATPase/helicase activity from this DDX3 core construct (V168-G582) is perhaps due to the lack of posttranslational modification, as it is produced in *E.coli*. Another possible explanation for the lack of ATPase/helicase activity would be the lack of the flanking amino- and carboxy- terminal. Other DExD box proteins also have decreased ATPase/helicase activity when the flanking regions are deleted [18, 19]. Recently, it was shown that indeed the N-terminal (135-168) of DDX3 harbors an ATP-binding loop, which interacts with ATP in an RNA-stimulated fashion [20]. This could have implications for our functional understanding of the flanking regions of DDX3X.

Shown in Fig. 2, DDX3X with AMP binding exhibits an open conformation. As P-loop has flexibility, DDX3X can adapt to several conformations. Because of the structural similarities with the protein Vasa [21], DDX3 will most likely obtain the closed conformation by rotating domain 2 approximately 180 ° relative to domain 1 with the addition of ATP and RNA binding [22].

### Translation regulation (mRNA metabolism)

DEAD-box proteins are involved in all steps of RNA metabolism, yet our knowledge about the RNA processing functions of DDX3 specifically continues to expand. DDX3 has been shown to be involved in promoter regulation of p21, E-cadherin, and IFN-β [23–25]. Further down the line of RNA processing, DDX3 has also been found in the exon junction complex [26] and mRNA export [27, 28]. Most importantly, DDX3 seems to function in translation initiation because of its interaction with 80S ribosomes and eukaryotic initiation factors [29, 30].

The various roles of DDX3 in RNA metabolism indicate a broad functional domain for DDX3. However, DDX3 knockdown did not affect general protein synthesis [28, 31, 32]. Instead, it has been suggested that specific co-factors [30, 33, 34] and RNA specificity [31, 35] determine the functionality of DDX3. DDX3 has been co-immunoprecipitated with eIF4E, PABP1, Ezrin, and eIF3, all part of a cap dependent translation initiation complex. It is hypothesized that DDX3 destabilizes complex RNA structures as part of the eIF4F translation complex to facilitate translation of specific mRNA’s with complex 5’-UTR’s [31, 35]. Also, DDX3 may be organizing translational control of cellular stress via stress granules.

### DDX3 and the stress response

The stress response in eukaryotic cells often inhibits translation initiation and leads to the formation of cytoplasmic RNA-protein complexes, referred to as stress granules. Stress granules serve as a reservoir of non-translating mRNAs, translation initiation components, and many additional proteins affecting mRNA function that allow the cell to respond quickly under stress conditions [36]. Stress granules play a protective role during stress by stalling general protein translation, allowing specific mRNA translation for adaptation and repair, and facilitation of post stress recovery by acting as reservoirs [37–39].

DDX3 has been found in stress granules and is involved in assembling these stress granules in an ATP-independent manner [28, 33]. Promoting stalled translation of stress-specific factors in an ATP-dependent manner by Ded1p [40, 41] is also most likely a function of the human orthologue DDX3. Gle1A is another protein involved in stress granule assembly and translation under stress conditions. Stress granules and translation defects, initiated by Gle1A knockdown, are rescued by expression of DDX3, underlining the importance of DDX3 in stress granule dynamics [42]. However, some factors need to be taken into consideration: First, not all stressors give rise to the same response in stress granule assembly or composition [43]. Secondly, stress granules may not always be involved in DDX3 related stress responses. But, at least DDX3 seems to be involved in the stress response to hypoxia and radiation, via modulation of apoptosis and cell cycle control [35, 44–47].

### Hypoxia

The process of tumor progression is characterized by rapid cellular growth, typically displaying a broad range of structural and functional abnormalities leading to tumor hypoxia [48, 49]. Hypoxia inducible factor-1 (HIF-1) is a transcription factor, key in cellular survival during hypoxia and is associated with tumor progression and metastasis in various solid tumors [50]. The DDX3 promoter has three HIF-1 responsive elements (HRE) [29] to which HIF-1α binds. Under hypoxic conditions HIF-1α promotes DDX3 expression through promoter activation at the most proximal HRE to the transcriptional start site [46]. In breast cancer patient samples, DDX3 is strongly correlated to hypoxia markers, specifically HIF-1α. Interestingly, the correlation between proteins related to HIF-1α and DDX3 is usually observed in a PI-3 K/AKT dependent fashion [45].

### Apoptosis

The ability of tumors to grow is not only determined by the rate of cell proliferation, but also by the rate of cell death. Acquired resistance to apoptosis (i.e., programmed cell death) is a hallmark of most and perhaps all types of cancer [51]. Apoptosis can be categorized as intrinsic (mitochondrial) or extrinsic (death receptors), but there is considerable crosstalk, finally leading to activation of effector molecules like caspase 3, caspase 7, or PARP.

Stimulation of death receptors (extrinsic apoptosis) causes receptor trimerization, followed by recruitment of Fas Associated with Death Domain protein (FADD) and caspase-8 to form the death-inducing signaling complex (DISC) [52]. Using 2D gel shift assays and mass spectrometry, DDX3 was identified as a TRAIL-R2 (death receptor) associated protein. TRAIL-R2 signal transduction involves the disassociation of DDX3, which counterbalances death signals [53]. Several proteins are known to provide protection from apoptosis, but few are known to act specifically at death receptors to inhibit apoptosis. A few known examples are cellular FLICE inhibitory protein c-FLIP [54], cellular inhibitor of apoptosis protein-1 (cIAP-1) [55], and Glycogen synthase kinase-3 (GSK3) [56]. DDX3 is also involved in forming death antagonizing signaling complex with GSK3 and cIAP-1 at each of the four major death receptors (Fas, TNF-R1, TRAIL-R1, and TRAIL-R2) thus inhibiting apoptotic signaling. Strong stimulation of death receptors overcomes this antiapoptotic complex by inactivating GSK3 and cleaving DDX3 and cIAP-1, permitting progression of the apoptotic signal [57]. Impairment of the death receptor-induced disabling of these proteins contributes to the evasion of apoptosis, specifically in triple negative breast cancer (lack of ER, PR and HER2 expression) [57, 58]. The relationship between DDX3 and the death receptor complex is currently tested in patients with metastatic triple negative breast cancer. DDX3 will be assessed as a predictive biomarker for TRAIL-R2 treatment as part of a phase II clinical trial; results are expected May 2017 (ClinicalTrials.gov NCT01307891).

It was reported that DDX3 functions irrespective of p53 however, all hepatocellular cancer samples with enhanced DDX3 mRNA expression also harbored p53 mutations [24]. In contrast with these results, in lung cancer, it was shown that p53 inactivation (HPV or mutation) reduced DDX3 expression by transcriptional regulation [59, 60]. P53 responds to the nature and extend of many stressors in different tissues very differently [61, 62], perhaps this could explain some of the contradictory outcomes with respect to DDX3. For instance, following DNA damage, DDX3 regulates apoptosis in a p53-dependent manner. In cells expressing wild-type p53, DDX3 associates with p53, increases p53 accumulation, and positively regulates camptothecin-induced apoptotic signaling via activation of caspase 7. Paradoxically, in cells expressing mutant or non-functional p53, DDX3 inhibits apoptosis by reducing caspase 3 activation [44]. The exact mechanism by which DDX3 affects cell fate is not clear, especially with respect to p53.

### Cell cycle regulation

To maintain genome integrity, cells need to adequately respond to various modes of genotoxic stress. This is achieved by activation of evolutionarily conserved DNA-damage response pathways that abrogate cell-cycle progression when the genome is damaged and stimulate DNA repair. Depending on the extent of DNA damage, cells either manage to repair all lesions and re-enter the cell cycle (checkpoint recovery), or are eliminated by apoptosis. Alternatively, cells can remain permanently arrested after a DNA-damaging insult (senescence) [63].

To balance the cellular stress response, DDX3 is essential to conserve cell cycle progression. Knockdown of DDX3 expression reduces growth and proliferation, most likely by impeding the G1/S-phase transition of the cell cycle through cyclin D1 and cyclin E1 mRNA translation [4, 23, 30, 32, 35, 64–68]. In contrast to the role of DDX3 in the stress response as described above, the H Lee group and Y-H Wu Lee group have reported that DDX3 reduces cell cycle progression via p53-DDX3-p21 regulation [24, 59, 69]. Perhaps, the HPV-, HBV-, and HCV-related nature of their tumor models has a distinct effect on DDX3 function in cancer. This is probable, as DDX3 is an important co-factor in the pathogenesis of these viral diseases [15]. The preserved PL-10 gene (DDX3 homologue) in the mouse fibroblast cells (NIH-3 T3) that were used could also complicate matters. Furthermore, the ambiguous role of DDX3 in regulating p21 could partly explain the discrepancies [23, 65]. Lastly, DDX3 facilitates translation of specific sets of mRNAs, but when overexpressed, suppresses general translation [33, 69]. Collectively, the effector functions of DDX3 in different cell types and the differential effects observed following DDX3 manipulation may explain the variable results observed.

### Wnt regulation

The Wnt signal transduction cascade is involved in developmental processes and various diseases, including colon cancer, medulloblastoma, and melanoma [70]. The first indication of the involvement of DDX3 in Wnt signaling came from the closely related DEAD-box protein DDX5. Like the epithelial-mesenchymal transition that occurred after overexpression of DDX3 in breast cancer cells [23], phosphorylated DDX5 interacted with nuclear β-catenin and subsequently stimulated EMT via a Wnt-independent pathway [71]. Through a whole-exome hybrid capture and with deep sequencing, activating mutations of DDX3 have been found in the majority of Wnt-driven medulloblastoma tumors. Moreover, mutant DDX3 potentiates transactivation of the TCF promoter and enhances cell viability in combination with mutant, but not wild-type, β-catenin [72]. Recently, three papers described the interaction with DDX3 and the Wnt signaling cascade. Xenopus and C.elegans development depends on Wnt signaling, which was impaired by knockdown of DDX3. They found that DDX3 acts as a V-type allosteric activator of CK1ε activity, to phosphorylation disheveled and thereby activating β-catenin, in an ATP hydrolysis and helicase-independent fashion [73]. Another mechanism in which DDX3 can impair Wnt signaling is via translational control of Rac1. They show that β-catenin is stabilized in the presence of Rac1 to increase Wnt signaling. Likewise other proteins with a complex 5’-UTR, Rac1 also depend on DDX3 for efficient translation. Hence, translational regulation of Rac1, by DDX3, does not only result in cytoskeletal remodeling but also affects Wnt regulation [74]. This is all in line with our paper in which we reported that loss of DDX3 function, by shRNA or DDX3 inhibitor, impaired Wnt signaling and caused disruption of the DDX3- β-catenin axis in lung cancer. However, we also show that DDX3 binds directly and co-localizes to β-catenin, which is not necessarily in line with either the DDX3-CK1ε-β-catenin or the DDX3-Rac1-β-catenin pathway [47]. Also, since the DDX3 inhibitor RK-33 was designed to bind to the ATP binding domain, it is unlikely that this goes via the ATPase and helicase independent DDX3-CK1ε-β-catenin pathway. Further research is warranted to elucidate the role of DDX3 in Wnt signaling.

### DDX3 in human samples

The differential expression of DDX3 and its orthologous *in vivo* and *in vitro* systems has greatly informed us about the functionality of DDX3. To translate these findings to a clinical setting, it is important to understand the expression, distribution, and regulation of DDX3 in cancer patients. Herein we describe the different findings of DDX3 in human cancer samples, as related to its utility as a prognostic and predictive biomarker, and its role in cancer biogenesis.

### Protein expression

In several cancer types, DDX3 expression has been evaluated in pathological samples. The variability in antibody usage, scoring of nuclear and/or cytoplasmic staining, and study population led to several differences (Table 1). Initially, it was reported that mRNA levels of DDX3 is elevated in 64 % of liver cancer patients [65] and 52 % of glioblastoma patients [75]. On the other hand, Y-H Wu Lee’s group found a decrease of DDX3 levels by qPCR and immunohistochemistry (IHC) in most liver cancer patients (50-73 %) and a positive association with p21 [24, 68]. Interestingly, in squamous cell carcinomas of the skin they found reduced *nuclear* expression whilst their illustrative pictures also suggest a *cytoplasmic* increase of DDX3 expression [24]. These interesting results led to more dedicated biomarker studies in human cancer samples.

**Table 1:** DDX3 expression in cancer patients

			DDX3 (% of samples)						
					protein				
Cancer type	subtype	study size (n)	DNA (mutated)	RNA (high expression)	nuclear	cytoplasmic (high expression)	prognostic value	other findings	ref
Breast cancer	all	366	-	-	20 %	35 %	HR 2.01 (95 % CI; 0.99–4.08)		[45]
	all	152	-	-	-	45 %	HR 2.06 (RNA-seq)*		[79]
Lung cancer	predominantly non-smokers	144	-	47 %	-	53 %	HR 0.62 (95 % CI; 0.40–0.96)	DDX3 association with:	[59, 76]
								E-cadherin (OR=3.32; p=0.007), p21 (OR=3.25; p=0.001), HPV (OR=0.30; p=0.002)	
	predominantly smokers	95	-	-	5 %	66 %	HR 2.10 (95 % CI; 1.13–3.93)		[47]
Colon cancer		221	-	-	-	60 %	HR 0.45 (95 % CI; 0.31–0.65)	inverse association with metastasis (RR=0.44; p=0.005)	[79]
		303	-	-	-	41 %	-	DDX association with nuclear β-catenin (RR=1.77; p<0.001)	[92]
Gallbladder cancer		126	-	-	-	55 %	15 months (low DDX3) vs 7 months (high DDX3)#		[81]
Liver cancer		45	-	64 %	-	-	-		[65]
		26	-	9 %	-	4 %	-		[24]
		41	-	-	-	19 %	no prognostic significance		[68]
Head and neck cancer	oral squamous cell cancer	324	-	-	11 %		HR 0.23 (95 % CI; 0.07–0.75) – non-smokers	decrease of nuclear expression, increase of cytoplasmic expression in dysplastic epithelium	[77]
					(nuclear or cytoplasmic)		HR 1.12 (95 % CI; 0.41–3.04) – smokers		
	oral squamous cell and oropharyngeal cancer	423	-	-	-	51 %	HR 0.88 (95 % CI; 0.53–1.45) – non-smokers		[78]
							HR 1.34 (95 % CI; 1.00–1.81) – smokers		
	oral squamous cell cancer	107	-	-	-	47 %	no prognostic significance		[79]
	all	74	4 %	-	-	-	-	not evidently HPV dependent, mutations in oropharyngeal cancer	[84]
	HPV+	51	8 %	-	-	-	-	probably loss of function mutation	[90]
	HPV-	69	0 %	-	-	-	-		[90]
	oral squamous cell carcinoma	50	10 %	-	-	-	-	homozygous deletions, not evidently HPV dependent	[91]
Skin cancer	squamous cell cancer	34	-	-	decreased nuclear and increased cytoplasmic DDX3 expression compared to normal		-		[24]
Brain cancer	medulloblastoma	92	8 %	-	-	-	-	probably gain of function mutation, association with WNT subtype (50 % of WNT subtype has DDX3 mutation)	[72]
	glioblastoma	31	-	-	52 % (western blot)		-	association between DDX3 and snail (p=0.001)	[75]
Leukemia	chronic lymphocytic leukemia	91	3 %	-	-	-	-		[85]

* = but no prognostic significance in microarray or IHC

# = average

HR = hazard ratio; OR = odds ratio; RR = relative risk; 95 % CI = 95 % confidence interval

In a Taiwanese cohort of 144 lung cancer patients, DDX3 is found to be a positive prognostic factor on overall survival, hazard ratio (HR) = 0.62 (95 % CI; 0.40–0.96). Moreover, DDX3 has a positive association with E-cadherin (OR = 3.32; p = 0.007) and p21 (OR = 3.25; p = 0.001), and a negative association with HPV (OR = 0.30; p = 0.002) [59, 76]. This contrasts with a Dutch cohort of 95 lung cancer patients, where patients whose lung cancer samples expressed high levels of DDX3 died on average 18 months earlier compared to patients with low DDX3 expressing tumors (HR = 2.10; 95 % CI; 1.13–3.93) [47]. In the Taiwanese cohort by Wu et al., 28 % of patients had HPV related lung cancer and less than 40 % of patients had a history of smoking [59], whereas most patients were smokers in the Dutch cohort. Perhaps this could explain, at least in part, the difference found in prognosis related to DDX3 expression.

In 324 oral squamous cell carcinoma (OSCC) patients, DDX3 was assessed by pooling nuclear and cytoplasmic expression and scoring whichever was the highest. This showed to be a positive predictor for survival (HR = 0.42; 95 % CI; 0.20–0.89). This is somewhat surprising since in the same paper it is reported that cytoplasmic DDX3 expression increases and nuclear DDX3 expression decreases in dysplastic oral epithelium [77]. Interestingly, we showed an inverse relation between cytoplasmic DDX3 expression and survival rate in 291 oral squamous cell carcinomas of smoking patients (HR = 1.34; 95 % CI; 1.00 - 1.81) [78]. Again, patients with oral squamous cell carcinoma in the Taiwanese cohort by Lee et al. are mainly non-smokers and HPV positive, whereas patients in the Dutch cohort were mainly smokers. Both in lung cancer and in oral squamous cell carcinoma, differences in survival related to DDX3 expression seem HPV/smoking dependent, however the underlying mechanism is yet unclear.

In 366 breast cancer patients, cytoplasmic DDX3 was increased, showed a correlation with the hypoxia response [45], and had an overall worse survival (HR = 2.01; 95 % CI; 0.99-4.08). In public databases of RNA expression in cancer, high DDX3 level was a poor prognostic indicator in RNA sequencing analysis but not in microarray analysis (HR 2.06; p < 0.001). In this same publication, the authors could not establish a correlation between survival and DDX3 protein expression in breast cancer, due to the limited power of the study [79]. Interestingly, it was suggested that women express higher levels of DDX3 since it is located at chromosome X and escapes X-inactivation in women [80].

In a cohort of 221 colon cancer patients, DDX3 expression was shown to be a positive predictor for survival both at the RNA and the protein level (HR = 0.45; 95 % CI; 0.31-0.65) [79]. For colon cancer specifically, the high frequency of both mutations in p53 and dysregulation of the Wnt pathway, in combination with the earlier described association of DDX3 with these oncogenic pathways, may explain why there appears to be a positive prognostic association with DDX3. This might not be the case in specific sub groups.

In gallbladder cancer as well, high DDX3 expression was related to worse survival both in squamous cell carcinoma, 13 vs 8 months (p = 0.003), as in adenocarcinoma, 15 vs 7 months (p < 0.001) [81].

The localization of DDX3 within the cell might determine its different functions. In general, DDX3 appears to accumulate in the cytoplasm of the cell. But, there are also reports of nuclear localization of DDX3 in HeLa cells and liver tissue, as well as the suggestion of a shift from nuclear to cytoplasmic localization during tumor progression [24, 77]. Because RNA transcription and translation occur in two discrete compartments, eukaryotic cells must evolve highly efficient mechanisms to traffic macromolecules such as RNA into and out of the nucleus. DDX3 proteins are exported from the nucleus to the cytoplasm in combination with chromosome maintenance region 1 (CRM1) [27] and Tip-associated protein (TAP) [28].

The diverse conclusions of these studies can be explained by the variability in antibody usage, localization of DDX3 (nuclear and/or cytoplasmic), the presence of virus mediated cancer (HBV, HCV, HPV), smoking status, and cancer type. However, the extent and molecular mechanisms of these factors that contribute to the cancer biogenesis process requires further investigation.

### DDX3 and metastasis

In support of the role of DDX3 in promoting metastasis, Chen et al. [74] showed that loss of DDX3 decreases Rac1 and β-catenin proteins, leading to lower Wnt/β-catenin target proteins. The functional consequences of this dysregulation are increased cell-cell adhesion and decreased cell motility and migration. We obtained similar results which demonstrated that knockdown of DDX3 in cancer cells decreased metastatic load in the lungs in a preclinical model of breast cancer [82]. Another key regulator of cancer metastasis, Ezrin, appeared to interact with DDX3 to control the translation of proteins involving the metastatic phenotype [34].

Besides perturbing intracellular signaling, DDX3 has been shown to induce epithelial-mesenchymal transition via Snail. Snail is a transcription factor that plays an important role in regulating cancer progression, especially invasion and metastasis [83]. By supporting increased cellular Snail levels, DDX3 influences cell proliferation and motility in a GSK3- and p53-independent fashion [23, 75]. This could be a potential mechanism of the increased propensity of metastatic disease in DDX3-overexpressing tumors [78, 81], although this was not shown in lung cancer or colon cancer [76, 79]. Here it was shown that loss of DDX3 also led to a loss of E-cadherin as a possible explanation for the increase in metastatic events. Collectively, this work supports the role of DDX3 in promoting metastasis.

### Mutations in DDX3

Through an improved understanding of the genetic basis of DDX3, we anticipate that future patients will be stratified and treated according to the biological makeup of their disease. DDX3 has been found as a driver mutation in a small set of head and neck tumors (4 %) [84] and chronic myeloid leukemia (CML) (3 %) [85].

Integrative genomic studies have recently identified at least four distinct molecular subgroups of medulloblastoma – Wnt, sonic hedgehog (SHH), Group 3, and Group 4 – which exhibit highly discriminate transcriptional, cytogenetic, and mutational spectra, in addition to divergent patient demographics and clinical behavior [86]. After CTNNB1, DDX3 is the second most frequently mutated gene in medulloblastoma (8 %). As much as 50 % (16 of 32) of all Wnt associated medulloblastomas and 11 % (7 of 66) of all SHH associated medulloblastoma cases harbored mutations in DDX3 [87]. Through mapping of the mutations to its crystal structure, it seems that the mutations alter DDX3 – RNA binding and are likely to result in altered protein function, as opposed to loss of function [20]. DDX3 mutations enhance cellular proliferation by potentiating the transactivation capacity of mutant β-catenin. Moreover, DDX3 is required to maintain the lineage of lower rhombic lip progenitor cells (origin of Wnt medulloblastoma cells) [72, 88, 89].

In head and neck cancer, missense mutations in DDX3 do occur but, the majority of genetic alterations are homozygous deletions, frame shift-, and nonsense- mutations, which is more supportive of loss of function. Interestingly, all mutations were found in oropharyngeal cancer [84, 90] and deletions were found in oral squamous cell cancer [91]. The influence of smoking and HPV probably determines the biology of the tumor and therefore determines the role that DDX3 plays in those tumors. One study found DDX3 to be exclusively mutated in HPV-positive HNSCC [90].

By mining the COSMIC database, we found only 12 % of genetic abnormalities of the DDX3 gene typical for tumor suppressor genes (nonsense mutation, deletions, frame shift or loss of heterozygosity), whereas 81 % of DDX3 genetic abnormalities are more typical for a gain of function (substitution missense mutations). In conclusion, DDX3 mutations are found in different types of cancer and seem to induce altered protein function rather than a loss of function. The extent to which these mutations impact clinical care by potential DDX3 inhibitors is yet to be determined.

### Functional divergence of DDX3-a potential tumor suppressor gene

In addition to the supporting evidence that DDX3 could act as a putative oncogene[23], there is an opposing view that loss of DDX3 promotes growth and could have potential tumor suppressor functions [24]. This is evident from publications [24, 59, 68, 76], which indicate that DDX3 is a transcriptional activator of p21 and is directly regulated by p53. Furthermore, the Lee group showed that loss of P53 decrease DDX3 expression, thus promoting tumor malignancy via the MDM2/Slug/E-cadherin pathway [76]. This is in contrast with others who indicate that DDX3 does not activate p21 [23, 45] and loss of DDX3 impairs growth and proliferation [44]. Furthermore, a recent paper showed that loss of DDX3 expression promotes metastasis in colorectal cancer [79]. Interestingly, our recent publication indicates that DDX3 expression is associated with aggressive colorectal cancer [92] as well as in sarcomas [93]. Of note is a paper by Jiang et al. [94] that identified mutations in DDX3 as potential drivers of natural killer/T-cell lymphoma. Given the diametrical opposite functions of DDX3 in cancer biogenesis, what could be the potential mechanisms of this dual role of DDX3? A study on the role of the Saccharomyces cerevisiae DDX3 homolog, Ded1, has suggested that it can act both as a repressor of translation initiation through its ability to interact with other components of the translation initiation factors and as an activator via its ATP-dependent activity [40]. Also, one can posit that DDX3 functions in a temporal fashion, exhibiting both an unstable and a stable phenotype. Given that tissue culture experiments can be viewed as a snapshot data collection point, it is crucial to define the end-points of each experiment. Moreover, patient sample variation and heterogeneity of the molecular pathogenesis of the diverse cancer types in different continents may contribute to the oncogenic/tumor suppressor functions of DDX3. For example, it is possible that in a sub-set of patients with a concurrent dsDNA viral infection (HPV, HBV, HCV), the role of DDX3 can be alerted [68]. Also, the different populations may have different stochastic ratios of co-factors associated with DDX3 that might facilitate differential functions of DDX3. At the present time, it is only speculative and requires a concerted effort by all the investigators in this field to delineate the biological role of DDX3 in the context of cancer biogenesis.

### Inhibitors of DDX3

DDX3 inhibitors were initially designed for treatment of human immunodeficiency virus-1 (HIV-1) [27, 95–97]. Small molecule inhibitors against DDX3 were discovered through rational design and by high throughput screening of commercially available compounds. The high throughput docking screen is based on the pharmacophoric model of the X-ray crystallographic structure of DDX3 in complex with AMP [17] as a three-dimensional filter to screen in sillico databases of compounds targeting the ATP binding site of DDX3. GoldScore and ChemScore were applied for scoring how fit the potential ligands bind to the ATP binding site [98]. According to the X-ray crystallographic structure of DDX3 in complex with AMP [17], the interactions between DDX3 and AMP consist of six hydrogen bond interactions and one π-π interaction (Fig. 3). While a general review on helicase inhibitors was published recently [99], this section focuses on DDX3 inhibitors as anticancer drugs.

**Fig. 4:**
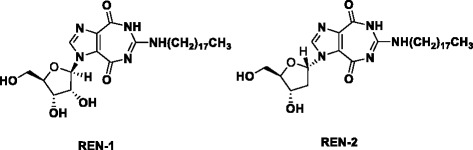
Structure of ring-expanded nucleosides targeting DDX3, REN-1 and REN-2 [96]

Inhibition of helicase-catalyzed ATP hydrolysis can logically be done by nucleotide and nucleobase analogues. As such, ring expanded nucleoside (REN) analogues, which structurally mimic adenosine nucleoside, inhibit helicase activity of DDX3 (structures shown in Fig. 4) [96]. One of the REN analogs studied in our laboratory exhibited inhibition of cell motility and viability in breast cancer [82]. Based on the molecular model of DDX3, these REN analogues were structurally modified into a series of tricyclic 5:7:5-fused diimidazo[4,5-d:4’,5’-f][1, 3]diazepine analogues [47, 64, 100, 101].

From databases of commercially available compounds, rhodanine analogues, triazine derivatives, and diphenyl analogues were identified by virtual screening to bind to the ATP binding site of DDX3 [97, 98, 102]. Ten out of the original 70 entries were tested with increasing concentrations of inhibitors and variable ATP concentrations for Ki value, resulting in inhibition of the ATPase activity of human DDX3 by a rhodanine analogue (1a in Table 2) with IC_50_ of 5.4 μM. Modifications of the hit rhodanine analogue 1a were made for structure-activity relationship studies (Structure 1-3 in Table 2). Triazine-based analogues (5a) were also structurally modified and tested for Ki value against DDX3 ATPase activity (Structure 4-6 in Table 2). The cytotoxicity study indicates that these drugs possess potential anti-cancer properties [98]. It will be exciting to see further target validation and *in vivo* activity of these potential DDX3 inhibitors as anticancer drugs.

**Table 2:** Inhibitors of DDX3Original table attached in the e-mail.  Please use this  for the paper. Font and Bold features should be maintained

Structure for modification	Cmpd #	n	R1	R2	Ki [μM]		Reference
	1a	2	2-OH	3-Br		5.4	
	1b	2	2-OH	3-F		0.3	
	1c	2	2-OH	3,5-diF		0.5	
	1d	2	2-OH	3-(O-CH_2_-O)-4		1.0	[98]
	1e	2	2-OH	3,4,5-triOMe		0.1	
	1f	2	2-Cl	3,4,5-triOMe		3.9	
	1 g	2	2-COOH	3-(O-CH_2_-O)-4		0.4	
	1 h	2	2-COOH	4-OMe		2.0	
	2a	2	2-OH	3-Br		4.2	[98]
	2b	2	2-OH	3-F		4.3	
	3	2	2-OH	3-Br		28	[98]
Structure for modification	Cmpd#	n	R1	R2	R3	Ki [μM]	
	4a	0	NH-Ph	H	-	0.4	
	4b	0	morpholinyl	3-Cl	-	1.6	[98]
	4c	1	morpholinyl	3-Cl	-	2.9	
	4d	1	NEt_2_	H	-	0.1	
	5a	0	NH-Ph	H	Ph(4-NHCOCH_3_)	0.3	
	5b	1	NH-Ph	H	Ph(4-NHCOCH_3_)	0.5	
	5c	0	morpholinyl	H	Ph(4-NHCOCH_3_)	2.2	
	5d	0	morpholinyl	F	Ph(2-OH)	0.7	
	5e	1	morpholinyl	H	Ph(2-OH)	0.6	[98]
	5f	1	morpholinyl	H	Ph(2-OH, 5-Cl)	1.9	
	5 g	1	morpholinyl	H	Ph(2-OH, 3-NO_2_)	4.0	
	5 h	0	morpholinyl	4-F	Ph(2-OH)	0.4	
	5i	0	NH-Ph(4-F)	4-F	Ph(2-OH)	0.1	
	5j	0	morpholinyl	4-F	2-methyl-indol-3-yl	0.2	
	6a	0	morpholinyl	4-F	Ph(2-Cl, 4-NO_2_)	0.3	
	6b	0	piperidinyl	H	Ph(4-Cl)	0.4	[98]
Structure modification	Compound Name						
	RK-33						[47]
	ZINC00011012						[104]

**Fig. 5:**
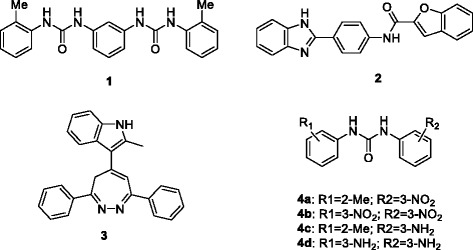
Inhibitors of DDX3 helicase function

DDX3 inhibitors, targeting the RNA-binding site of DDX3, were discovered by screening 220,000 entries of the Asinex database by high throughput docking [103]. The 3D structure of DDX3 in the closed conformation is currently unavailable. Thus, individual domains of DDX3 crystallized with AMP in an open conformation were aligned with corresponding domains of the closed conformation of the DEAD-box helicase eIF4A. Three lead compounds were identified (Compound 1, 2, 3 in Fig. 5). Structural modifications of 1 were made to increase binding affinity at the RNA binding site of DDX3 (4a-d in Fig. 5). Two compounds showed strong inhibition against DDX3 helicase activity (IC_50_ 4a = 1 μM ± 0.2 and 4c = 5 μM ± 0.6) [103].

**Fig. 6:**
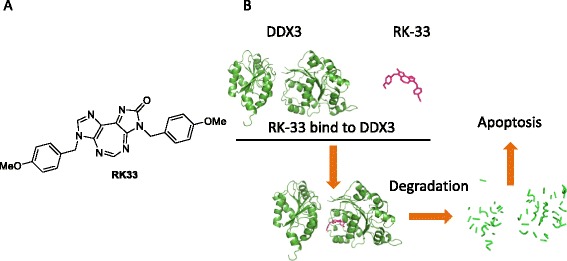
DDX3 inhibitor RK-33. **a.** Structure of 5:7:5 tricyclic heterocycle RK-33. **b.** Graphic depiction of the interaction of DDX3 and RK-33 and the subsequent biological effect

Recently, we reported a tricyclic diimidazodiazepine analogue, also known as RK-33, as a DDX3 inhibitor [47, 64, 101]. RK-33 is a derivative of REN analogues with a third ring added to the 7-member diazapine ring (Fig. 6a). RK-33 binds to DDX3, inhibits DDX3 helicase activity and cancer growth, and radiosensitizes lung cancer cells in a DDX3-dependent manner (Fig. 6b) [47]. A number of tricyclic diimidazodiazepine analogues were generated by structure modifications of RK-33. These compounds maintain their cytotoxic activity against breast, prostate, and lung cancer cell lines [47, 101]. In particular, the imidazolone ring could be altered to improve cytotoxicity [101].

Several compounds that inhibit DDX3 were identified through high throughput screening, resulting in the following: rhodanine derivatives and triazine analogues to bind to the DDX3 ATP binding domain, and biphenyl analogues to inhibit RNA helicase activity. These analogues have been investigated for their anti-HIV properties and their anticancer properties are under investigation. Conversely, the tricyclic heterocyclic RK-33 has shown potent anticancer activity against multiple cancer cell lines including, but not limited to, those of, sarcoma, and lung [47, 93]. The validation of DDX3 helicase activity inhibition by RK-33 was performed by measuring the proportion of unwound RNA duplexes (FAM-labeled) by DDX3 homolog Ded1p (80 nM) and varying concentrations of RK-33 [47]. Moreover, specificity assay of RK-33 binding to DDX3 but not the closely related protein DDX5 and DDX17 were also performed by pull-down assay of biotinylated RK-33 [47]. Recently, another compound called 5-benzoyl-2,3-dihydro-1H-pyrrolizine-1-carboxylic acid, tris (hydroxymethyl) amino methane salt (ZINC00011012) was explored as a DDX3 inhibitor in preclinical models of oral cancer [104]. Inhibition of DDX3 ATPase activity by ZINC00011012 was validated by measurement of Pi release from ATP in the mixture of purified His-DDX3 (6 μM) and varying concentrations of ZINC00011012 [104].

Predictive biomarkers are common practice with modern targeted therapies like anti HER2, ER, BCR-ABL, or EGFR treatment [105]. DDX3 expression level could serve as a predictive biomarker for clinical applications of targeted treatment. DDX3 inhibitors, with more validation, would be a new and promising strategy in tackling cancer.

## Conclusions

In this review, we have thoroughly examined the role of DDX3 in cancer. DDX3 is a DEAD-box helicase located on the X-chromosome with various roles in immunology and cancer. Functionally, DDX3 is specifically involved in promoter regulation, the exon junction complex, mRNA export, and translation initiation. During cellular stress, DDX3 can assemble stress granules in an ATP-independent manner or promote stalled translation of stress-specific factors in an ATP-dependent manner. DDX3 appears to be involved in at least the stress response to hypoxia and radiation, via modulation of apoptosis and cell cycle control.

Whether DDX3 has tumors suppressing abilities or facilitates the maintenance of the oncogenic state has been of considerable debate. Some state that DDX3 acts as a tumor suppressor gene by regulating p21 [24, 59, 68, 69, 76, 77, 79]. However, a majority has shown a plethora of different functions of DDX3, which enable a cancer cell to survive in an unstable state [23, 35, 44–47, 53, 57, 72, 73, 75, 81, 85, 88, 89, 93, 106–109].

Altogether, DDX3 is essential in maintaining cancer cell viability in non-virus mediated stress response by controlling the cell cycle and apoptosis. As a result, there is increasing effort to design or identify, via high throughput screening, new DDX3 inhibitors [97, 98, 102, 103]. Pre-clinical and clinical evaluation of those compounds will shed light on the applicability of this new paradigm to block cancer progression via inhibition of DDX3. Understanding efficacy of these novel inhibitors and the potential use of DDX3 expression as an appropriate biomarker will need further scrutiny.

## Abbreviations

DEAD/H: Asp-Glu-Ala-Asp/His
RNA: Ribonucleic acid
AzFa: Azoospermia factor a
ATPγS: Adenosine 5’-[γ-thio]-triphosphate
ADP: Adenosine diphosphate
AMP: Adenosine monophosphate
Tyr: Tyrosine
Arg: Arginine
Gln: L-glutamine
Gly: Glycine
Ser: Serine, Lys, lysine
Thr: Threonine
HIF-1: Hypoxia inducible factor 1
HRE: HIF-1 responsive elements
PARP: Poly ADP ribose polymerase
DISC: Death-inducing signaling complex
FADD: Fas associated with death domain protein
TRAIL-R2: Tumor necrosis factor-related apoptosis-inducing ligand receptor 2
FLICE: Caspase-8/FADD-like interleukin-1beta-converting enzyme
cIAP-1: Cellular inhibitor of apoptosis protein-1
GSK3: Glycogen synthase kinase-3
HPV: Human papillomavirus
HCV: Hepatitis C virus
HBV: Hepatitis B virus
HIV-1: Human immunodeficiency virus 1
TCF: Ternary complex factor
CK1: Casein kinase 1
qPCR: Quantitative polymerase chain reaction
IHC: Immunohistochemistry
HR: Hazard ratio
OR: Odds ratio
OSCC: Oral squamous cell carcinoma
CI: Confidence interval
CRM1: Chromosome maintenance region 1, TAP, Tip-associated protein
CML: Chronic myeloid leukemia
SHH: Sonic hedgehog
COSMIC: Catalogue of somatic mutations in cancer
REN: Ring-expanded nucleoside
